# Venture Capital's Role in Driving Innovation in Cardiovascular Digital Health

**DOI:** 10.1016/j.jacadv.2024.100921

**Published:** 2024-04-10

**Authors:** Partha Sardar, Angela Lee

**Affiliations:** a Division of Cardiology, Columbia University Irving Medical Center/New York-Presbyterian Hospital, New York, New York, USA; b Columbia Business School, New York, New York, USA; c Eugene Lang Entrepreneurship Center, Columbia Business School, New York, New York, USA

**Keywords:** cardiovascular, digital health, venture capital

In the last decade, there has been remarkable growth in venture capital (VC) investment in digital health.^1^ Between 2010 and 2017, digital health investments surged by 858%, totaling over $41.5 billion in this decade. Digital health funding reached a record $9.1 billion in 2020, a 21% increase from the previous year. Cardiovascular (CV) disease startups raised over $20 billion since 2020.^2^ Factors influencing growth in VC funding for CV digital health include rising adoption of digital health tech, demand for cost-effective solutions, and the potential to enhance care quality.^2^^,^^3^ Despite the opportunities VC-backed startups offer to reshape health care, they also present risks. Recognizing its impact on medical innovations, CV health care professionals and policymakers should understand VC basics, including investment decision processes, potential patient impacts, and implications for CV practices. This viewpoint reviews major VC-backed CV digital health companies, exploring concerns and benefits tied to the rise of VC backed digital health startups.

## How does venture capital work?

VC firms raise funds from investors (high net worth individuals, family offices, institutional investors) to support early-stage companies with high growth potential. In return for their investment, these firms gain equity and wield influence over strategic decisions.^3^^,^^4^ While startup founders excel in their domain, they may lack the expertise to navigate company growth. VCs specialize in guiding emerging companies through pivotal phases, subsequent financings, and eventual exits, either through acquisition or an initial public offering. Successful exits enable venture capitalists to realize returns on their initial investments.

## Venture capital investments in cardiovascular digital health

VC has significantly driven the growth of numerous CV digital health startups, investing in technologies such as wearable devices, mobile health apps, telemedicine, and personalized medicine tools. The cost-effectiveness of developing smartphone apps and monitoring technologies has lowered market barriers. Table 1 delineates VC backed key players in the CV digital health landscape.^5^^,^^6^

**Table 1:** Large Venture Capital–Backed Cardiovascular Digital Health Company/Start-Ups

Digital Health Company	Capital Raised in Millions $	Products or Services
Remote ECG monitoring		
AliveCor	134.33	AliveCor develops a mobile electrocardiogram (ECG) device to enhance stroke prevention by detecting atrial fibrillation early. The device allows patients to monitor their heart health using smartphones, providing physicians with an instant analysis tool for detecting atrial fibrillation and other abnormal rhythm in an ECG.
BioTelemetry	$163.24	BioTelemetry, a wireless medical technology company, is dedicated to delivering health information to enhance quality of life and reduce care costs. The company offers cardiac monitoring services, focuses on manufacturing cardiac monitoring devices, and provides centralized cardiac core laboratory services. On December 18, 2020, Philips acquired BioTelemetry.
Nuubo	$17.18	Nuubo specializes in wearable wireless medical technology. It provides noninvasive, wireless monitoring technology for medium- and long-term tracking of physiological parameters.
Hemodynamic monitoring		
Sensydia	20.8	The Sensydia Cardiac Performance System (CPS) is designed to provide accuracy equivalent to gold standard measures on a single, automated, and noninvasive platform. This enables swift assessment of key metrics like ejection fraction (Food and Drug Administration approved in 2018), cardiac output, and pulmonary artery pressure.
Entegrion	31.46	Entegrion's viscoelastic coagulation monitor is an advanced viscoelastic coagulation monitor, offering rapid, real-time hemostasis assessment at the point of care.
Respirix/Cardiospire	1.3	Respirix created Cardiospire, a point-of-care monitoring device for congestive heart failure, providing a noninvasive cardiac monitor to detect high-fidelity stroke volume, cardiac output, and pulmonary artery pressure.
Digital stethoscope		
Eko	127.4	Eko's solutions feature artificial intelligence-enabled stethoscopes, combining advanced software and AI-supported analysis to enhance the detection of cardiovascular disease.
Cardiovascular telehealth		
Heartbeat Health	28.2	Heartbeat Health offers a virtual-first cardiology practice with a data-driven health care platform. It provides clinical services, such as same-day diagnostic reads, tele-visits, and heart care programs for patients with atrial fibrillation, vascular diseases, and heart failure.
Patient data analysis		
CorVista	87.19	The CorVista System is an advanced noninvasive cardiac diagnostic platform that combines mathematics, physics, and machine learning to reshape care pathways. This point-of-care solution enables physicians to assess heart function without the need for radiation, contrast agents, injections, fasting, or exercise.
Xenter	14.1	Xenter is developing a data-driven, AI platform that integrates wireless capabilities into essential devices for treating heart attack, stroke, and vascular disease. By incorporating novel technologies, Xenter enhances workhorse medical devices, collecting Physical Intelligence and wirelessly transmitting it to a global health care cloud.
Hypertension and weight loss management		
Noom	627.8	Noom utilizes AI, mobile technology, and psychology to assist individuals in adopting healthier long-term habits. With a subscription-based app, it tracks food intake and exercise habits, emphasizing behavior change and mental wellness.
Omada Health	449.72	Omada Health is a virtual care solutions and digital behavioral medicine company focused on preventing chronic conditions like heart disease and type 2 diabetes. It provides behavioral counseling based on insights from social networking, gaming, and behavioral sciences.
Hello Heart	138	Hello Heart is a health technology company specializing in cardiovascular health. It creates a device for testing blood pressure and blood sugar levels, assisting patients in tracking and monitoring their health, medication intake, and physical exercise through a dedicated mobile application.
Cardiac rehabilitation		
Movn Health	39.22	Movn Health is a telehealth company facilitating access to virtual cardiac rehabilitation for patients across multiple states post-heart attack or cardiovascular complications.
Cardihab	1.35	Cardihab specializes in digital cardiac rehabilitation. The company delivers a digital therapeutic solution for cardiac rehab through smartphone apps and web portals, allowing clinicians to provide convenient and flexible services.
Recora	20	Recora offers comprehensive cardiac recovery from the comfort of users' homes through a virtual cardiac recovery program, providing the tools and guidance necessary for restoring heart health.
Consumer heart monitoring		
Nanoleq	13.5	Nanoleq offers smart textile solutions for health monitoring, tracking parameters like heart rate, respiration rate, temperature, activity, posture, and more.
Binah.ai	13.65	Binah.ai offers an AI-based health monitoring platform that measures various biomarkers, including blood pressure, heart rate, heart rate variability, oxygen saturation, breathing rate, sympathetic stress, parasympathetic activity, and pulse-respiration quotient.
Other digital care		
Cadence	141	Cadence is a health care technology company providing a remote care management platform. It allows clinicians to remotely monitor patient vitals, offer personalized feedback via text messages after each reading, and conduct one-on-one telehealth visits when necessary.
Satelia	10	Satelia provides a remote monitoring solution for heart failure, along with an alert system to help the medical team identify patients displaying potential health deterioration.

The capital raised information is sourced from CB Insights/PitchBook as of November 2023.^5^^,^^6^ Companies in the cardiovascular digital health sector were identified based on the presence of “cardiovascular” and “digital” in their keywords and/or descriptions. Additional details were gathered from company websites. It's important to note that the provided list of companies is not exhaustive.

AliveCor, a VC-backed leader, raised 134.3 million for their credit card-sized medical-grade electrocardiogram devices. The remote patient monitoring space also benefits from favorable reimbursement dynamics by the Centers for Medicare and Medicaid Services. Other companies in remote patient monitoring, BioTelemetry and Nuubo raised $163.2 and $17.2 million, respectively, for their wireless medical technologies (Table 1).

The COVID-19 pandemic accelerated telehealth utilization as health care professionals restricted face-to-face visits. Remote delivery of information, diagnosis, disease monitoring, and follow-up care demonstrated significant changes specific to CV disease management. Heartbeat Health offers a virtual-first CV practice and has raised $28.2 million.

Weight loss programs by companies like Noom, raised $627.8 million, and offer personalized coaching, nutritional guidance, monitoring technology, and prescriptions for weight loss drugs. Hello Heart, secured $138 million, provides coaching along with comprehensive blood pressure monitoring and easy access to physicians. These digital solutions enhance traditional treatment plans by delivering personalized care outside clinical settings.

Companies like Movn Health, Recora, and Cardihab offer virtual cardiac rehabilitation programs and raised $39.2, $20, and $1.35 million, respectively.

## Potential advantages to venture capital investment

VC-backed startups catalyze CV digital health innovation by providing essential funding, support, and guidance. These companies prioritize rapid scalability to serve a broad population, maximizing return on investment.^1^^,^^4^ VC firms subsidize constant model iterations, fostering proof of concept, and refining evolving products or services. The market-driven, “survival of the fittest” approach ensures success only when patients or populations perceive benefits. VC-backed digital health companies excel in transparency with diagnosis, treatment processes, and costs, marking progress for CV health care.

## Concerns related to venture capital investment

In recent years, there has been a notable shift in VC investment in health care, raising concerns among physicians, caregivers, payers, and patients. VC firms can cause a founder to prioritize consumer and revenue growth without stringent quality assessments, potentially compromising health care standards.^1^^,^^4^ The pricing of startup technologies remains uncertain, and if improvements come at a higher total cost, policymakers and adopters face challenging decisions on access and tradeoffs. The uniformity of digital products for scalability may limit solutions for patients with comorbidities, aggravating health equity divides.^1^ Demonstrating efficacy in digital health interventions poses a challenge, as the VC reality of seeking quick returns can conflict with the imperative to ensure no harm. This trend underscores the need for careful consideration and policy frameworks to address potential pitfalls in the evolving landscape of VC-backed health care innovations.

## Challenges and opportunities

Digital health VC faces challenges, notably the high risk in early-stage startups, where expected milestones and returns may not materialize. Regulatory hurdles from federal agencies add complexity and cost to bringing digital health products to market. Despite these obstacles, the sector offers substantial opportunities. Multiple studies have reported several advantages of utilizing digital health to enhance health care quality, efficiency, and accessibility and improve care for patients with heart failure, arrhythmia, and coronary artery disease.^7^ Investing in digital health startups enables participation in the sector's growth. However, the total VC funding for CV solutions in digital health decreased by 31% from 2021 to 2022, totaling $3.4 B in 2022.^5^ This trend is similar to other sectors and can be explained by economic concerns, rising interest rates, and fewer exits that have impacted venture funds, thereby reducing the capital availability for new investments.^5^

## Future outlook

The evolving digital health care landscape is propelled by the impactful force of VC-backed for-profit companies. While the potential value of this trend remains to be fully realized, the surge in VC investment in CV digital health care presents an opportunity to scale effective solutions for widespread CV issues. However, substantial challenges like quality control, privacy concerns, and addressing severe CV illnesses require attention. To establish a trustworthy evidence base, publicly funded independent studies are essential, assessing the impact of VC-backed products on clinical and economic outcomes. VC holds vast potential for introducing innovative digital health solutions to address unmet medical needs. However, its efficacy is constrained by economic realities and incentives. With the recent surge in CV digital health venture investments, cardiologists should stay vigilant to the forces shaping new medical technologies and their implications for diagnostics and treatments in the field.

## Funding support and author disclosures

Dr Lee teaches venture capital, leadership, and strategy courses at Columbia Business School, and is a startup investor and the founder of 37 Angels, an investing network that has evaluated 15,000 startups, invested in 80, and activates new investors through a startup investment boot camp. Dr Sardar has reported that he has no relationships relevant to the contents of this paper to disclose.
